# Lymphoscintigraphy in patients with breast cancer-related lymphedema after sentinel lymph node dissection and axillary radiation therapy

**DOI:** 10.1097/MD.0000000000031985

**Published:** 2022-12-09

**Authors:** Se Hyun Oh, Ju Hyeon Kim, Seung Tae Seong, Jun Young Park, Jae Hyun Lee, Ghi Chan Kim, Ho Joong Jeong, Young Joo Sim

**Affiliations:** a Department of Physical Medicine and Rehabilitation, Kosin University Gospel Hospital, Busan, Korea.

**Keywords:** breast cancer lymphedema, lymphoscintigraphy, radiotherapy, sentinel lymph node

## Abstract

The purpose of this study was to investigate lymphoscintigraphy pattern according to the presence or absence of axillary site radiation therapy (aRTx) in breast cancer-related lymphedema (BCRL) patients who underwent sentinel lymph node dissection (SLND). The participants were patients who visited our facility from July 2014 to June 2021 due to upper extremity edema. Among them, patients who underwent SLND after the diagnosis of breast cancer were included. The participants were divided into a group without aRTx (group A) and a group with aRTx (group B). In each patient’s lymphoscintigraphy, axillary lymph node uptake (ALNU), lymphatic flow delay, dermal back flow, and the presence of any collateral pathway were checked. Thirty-three patients were enrolled. In all, 27 patients were classified in Group A, and 6 patients were classified in Group B. Between the 2 groups, we found a significant difference (*P* value < .05) between groups at ALNU and lymphatic flow delay. However, there was no significant difference between groups at the dermal backflow and the presence of a collateral pathway (*P* value > .05). And 24.2% of patients who developed lymphedema after SLND showed normal lymphoscintigraphy. In this study we suggest that SLND and aRTx affects the activity of the axillary lymph node and ultimately adversely affects lymphatic flow, becoming a risk factor for lymphedema. In addition, regardless of SLND or aRTx, lymphedema may eventually occur in the patient with normal lymphoscintigraphy.

## 1. Introduction

Breast cancer occurs in 20.6% of Korean female cancer patients and represents the highest rate of cancer incidence in Korean women. Various modalities, such as mammography, ultrasound, magnetic resonance imaging, and computed tomography, for the early detection of breast cancer have been developed.^[1]^ As a result, the early diagnosis of breast cancer has also increased.^[1,2]^ In patients with early-stage (T1, T2) breast cancer without axillary lymph node metastasis, less-invasive sentinel lymph node dissection (SLND) or mastectomy is preferred over axillary lymph node dissection (ALND).^[3]^ SLND is a surgical method in which 1 cc of 0.8% indigo carmine dye is injected intradermally in 4 directions around the areola before surgery to identify sentinel lymph nodes to determine whether to remove them according to the results of frozen sections.^[4]^ Compared to ALND, less-invasive SLND can reduce the incidence of postoperative complications, such as infection, axillary seroma, paresthesia, and breast cancer-related lymphedema (BCRL).^[5]^

Radiation therapy (RTx) lowers the local recurrence rate of breast cancer and increases the survival rate of patients.^[2]^ However, various complications can occur with RTx; upper extremity lymphedema is the most common.^[6–8]^ The incidence of BCRL in breast cancer patients who underwent SLND is 0% to 7%, which is lower than in those who underwent ALND.^[5]^ In addition, the incidence of BCRL increases when RTx is performed after surgery compared to cases when only surgery is performed; the incidence rate increases significantly when RTx is performed on an axillary site.^[7–9]^

Lymphedema is pathologically described as the accumulation of protein-containing fluid in the extracellular interstitium that exceeds the transport capacity of the reduced lymphatic vessels due to abnormalities in the lymphatic system.^[5,9]^ Lymphedema has many adverse effects on the quality of life of patients.^[10,11]^ Therefore, the Department of Rehabilitation Medicine at our facility is implementing complex decongestive therapy (CDT), such as manual drainage, bandaging, and pneumatic compression for these patients. In some patients, this treatment is effective, but in others it is not. In addition, due to the late diagnosis of lymphedema, the appropriate treatment time for lymphedema is often missed. Therefore, interpretation of lymphoscintigraphy is important for diagnosing lymphedema and predicting its severity.

Previous studies have examined the lymphoscintigraphy results in relation to BCRL in breast cancer patients who underwent surgery that included ALND.^[12]^ Lymphedema can also occur in patients who undergo SLND; to our knowledge, there have been no studies on lymphoscintigraphy in patients who underwent only SLND with or without RTx on an axillary site. In this study, we aimed to investigate the pattern of lymphoscintigraphy according to whether RTx was performed on an axillary site in BCRL patients who underwent SLND for breast cancer.

## 2. Material and methods

### 2.1. Participants

This study was a retrospective study that was conducted from July 2014 to June 2021 among people treated for BCRL who were inpatients or outpatients at Kosin University Gospel Hospital. All patients were diagnosed with unilateral breast cancer and underwent surgery; they visited the rehabilitation department with symptoms such as “swelling,” “heaviness,” and “numbness.” BCRL was diagnosed by a rehabilitation medicine specialist by synthesizing the following criteria: a difference between the circumferences of both arms of at least 2 cm or more from sites 6 cm distal, 12 cm proximal, 21 cm proximal, and 33 cm proximal from the styloid process; a difference between the volume of both arms at least 200ml or more (from finger tip to 33 cm proximal from the styloid process); a surgical history of breast cancer; skin conditions such as fibrosis; and self-reported symptoms.

The inclusion criteria are as follows: women over the age of 18, patients who completed 2 weeks of CDT, patients with a record of volumetric measurements before and after CDT, patients with lymphoscintigraphy, and patients who underwent only SLND excluding ALND.

The exclusion criteria were as follows: patients with signs of infection during hospitalization, patients diagnosed with vessel disease through Doppler ultrasound, patients with systemic edema, patients without volumetric measurements either before or after CDT, patients who did not complete 2 weeks of CDT, patients who were less than 3 months postoperative from their breast cancer surgery, patients with bilateral upper extremity edema, patients with a history of cancer other than breast cancer, patients taking medications that can cause fluid retention and extremity swelling, patients undergoing radiation or chemotherapy, patients with a history of diabetes, hypertension, cardiac disease, or kidney disease, and patients with insufficient medical records. This study was approved by the Institutional Review Boards (IRB No. 2022-06-042).

The patients were divided into a group without axillary RTx (aRTx) at supraclavicular lymph nodes and axillary lymph nodes (Group A) and a group with aRTx (Group B).

### 2.2. Complex decongestive therapy

All enrolled patients underwent CDT 5 times a week for 30 minutes at each time for a total of 2 weeks. CDT included manual lymphatic drainage, compression bandaging, and exercise education. All patients were treated by the same physical therapist.

### 2.3. Volumetric measurements

Volumetric measurements were performed both before and after CDT in all enrolled patients. The purpose of the volumetric measurements was to record the volume from the fingertips to 33 cm proximal from the styloid process using the water displacement method. All examinations were performed by the same rehabilitation physician.

The initial excess volume was defined as the difference in the volume of both arms before CDT. The reduction in excess volume was defined as the change in excess volume of the affected arm before and after CDT:


$$\begin{array}{l} Initial\;excess\;volume\;=\;V1a\;-\;V1u \end{array}$$

$$\begin{array}{ll} & Reduction\;of\;excess\;volume\\ & \;\;\;=\left(V1a-V1u\right)-(V2a-V2u) \end{array}$$

(V1a: affected arm volume before CDT, V1u: unaffected arm volume before CDT, V2a: affected arm volume after CDT, V2u: unaffected arm volume after CDT).

### 2.4. Bioelectric impedance analysis

All enrolled patients underwent bioelectric impedance analysis both before and after CDT. Bioelectric impedance analysis was performed after the patient had rested in a supine position for 10 minutes. The examination was conducted by placing the electrodes on the extremities, and the movement of the patient was restricted during the examination. Throughout the examination, the resistance values of both arms were measured at 1 kHz to calculate the ratio of the non-edematous arm to the edematous arm, and this was defined as the lymphedema index ratio (LIR). All tests were performed by the same rehabilitation medicine doctor using an Inbody S10ⓡ.


$$\begin{array}{l} LIR\;=\;\left(Z_{u}at\;1\;kHz\right)/(Z_{a}at\;1\;kHz) \end{array}$$

(Zu: impedance resistance of the unaffected arm, Za: impedance resistances of the affected arm).

### 2.5. Lymphedema stage

The lymphedema severity grade was classified as mild (Stages 0–I), moderate (Stage IIa), and severe (Stages IIb–III) using the International Society of Lymphology (ISL) staging system.^[11,13]^

### 2.6. ISL stages

0: A subclinical state where swelling is not evident despite impaired lymph transport.

I: Early onset of the condition where there is accumulation of tissue fluid that subsides with limb elevation.

IIa: Limb elevation alone rarely reduced swelling, and pitting is manifested.

IIb: There may or may not be pitting as tissue fibrosis is more evident.

III: The tissue is hard (fibrotic), and pitting is absent. Skin changes, such as thickening, hyperpigmentation, increased skin folds, fat deposits, and warty overgrowths, may develop.

### 2.7. Lymphoscintigraphy

**Figure 1.**
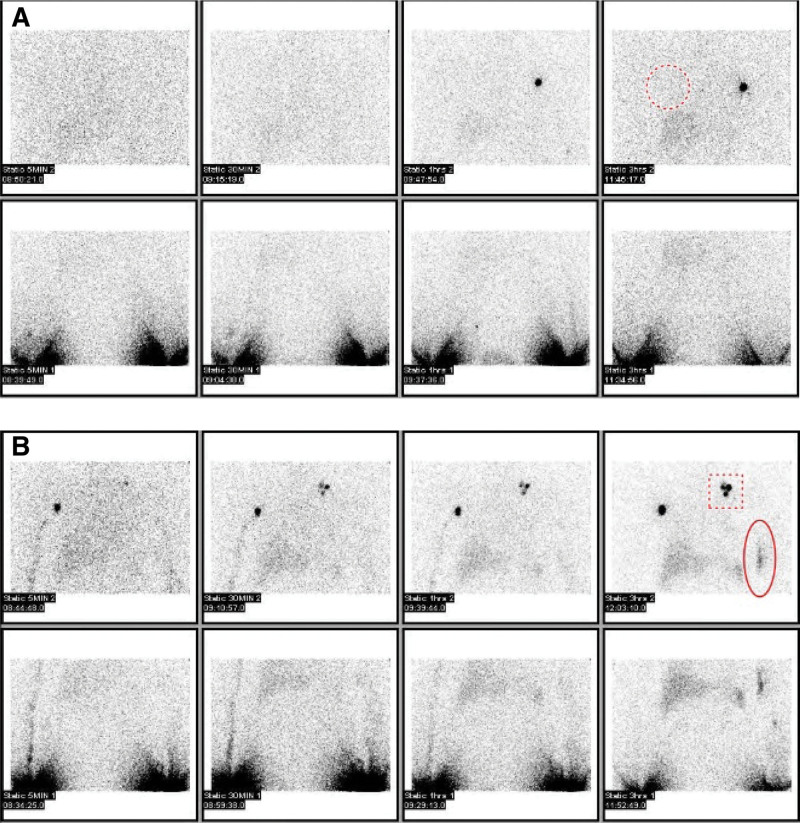
Abnormality at Lymphoscintigraphy. (A) Lymphoscintigraphy of a right breast cancer patient who underwent sentinel lymph node dissection. There are no axillary lymph node uptake on right side (dotted circle). (B) Lymphoscintigraphy of a left breast cancer patient who underwent left breast conserving surgery with sentinel lymph node dissection. There was dermal back flow (the dotted box) and a collateral pathway (oval) on the left upper limb.

**Figure 2.**
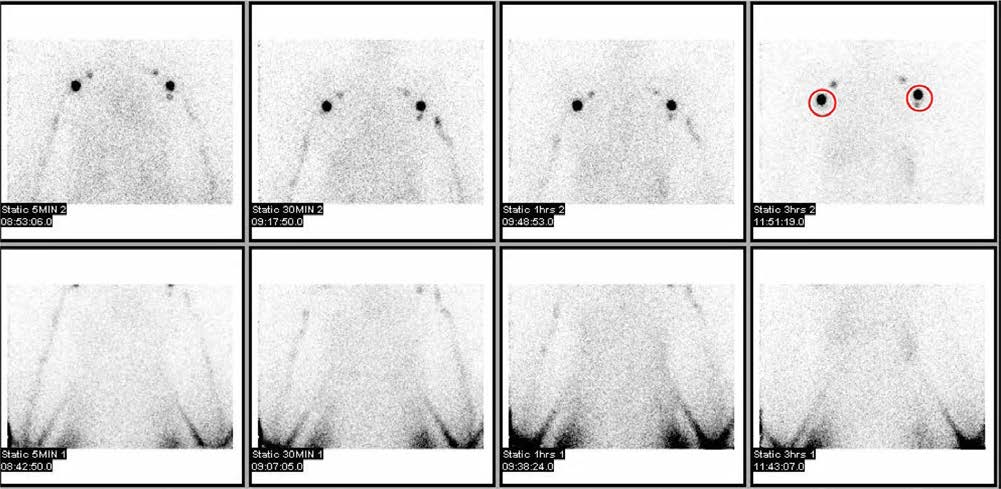
Normal lymphoscintigraphy. There are axillary lymph node uptakes on both side in circles.

Lymphoscintigraphy was obtained using an INFINIA model gamma camera manufactured by GM Medical Systems. After injecting 99mTC-phytate into the second web space (between the second and third fingers) of the hand, anterior-posterior and posterior-anterior views were obtained at 5 minutes, 30 minutes, 1 hour, and 3 hours after the injection. Lymphoscintigraphy was evaluated for axillary lymph node uptake (ALNU), lymphatic flow delay, dermal back flow, and the presence of any collateral pathways (Figs. 1 and 2).

### 2.8. Outcome measures

The primary outcomes were the lymphoscintigraphy findings of each patient and the differences between the presence of ALNU, lymphatic flow delay, dermal backflow and collateral pathway in the 2 groups. The secondary outcomes were differences in the volumetric parameter between the 2 groups, such as initial excess volume and the reduction of excess volume after CDT.

### 2.9. Statistical analysis

All statistical analyzes were performed using the Statistical Package for the Social Sciences, version 25.0 (SPSS, Inc., Chicago, IL). For the primary outcomes, Mann–Whitney *U* tests for ALNU, dermal back flow, and collateral pathways, while a Kruskal–Wallis test was used to evaluate the differences between Group A and Group B for lymphatic flow. The Student *t* test was used to compare the averages of each parameter of the 2 groups, including the secondary outcomes. *P* values < .05 were considered statistically significant.

## 3. Result

Initially, 46 patients were recruited, including 2 with bilateral upper extremity edema, 4 with lower extremity edema, 4 with a cancer history other than breast cancer, 1 with heart disease, 2 with vessel disease. Finally, 33 patients were enrolled in the study. In breast cancer patients who underwent SLND excluding ALND from July 2014 to June 2021, the incidence of BCRL was 2.5% in 58 out of 2320 patients.

The demographic data of each group are shown in Table 1. There were 27 patients (81.8%) in Group A and 6 patients (18.2%) in Group B. There were no statistically significant differences in height, weight, body mass index, LIR, RTx total volume in the patient characteristics between the 2 groups (Table 1).

**Table 1:** Generic characteristics of lymphedema patients.

Characteristics	Group A	Group B	*P* value
Total (n)	27	6	
Age (yrs)	56.77 ± 6.27	49.33 ± 7.20	.015*
Height (m)	1.58 ± 0.05	1.60 ± 0.05	.229
Weight (kg)	62.07 ± 7.59	60.25 ± 8.75	.608
BMI	24.93 ± 2.65	23.51 ± 3.70	.278
Affected arm side			
Rt. (n)	11	3	
Lt. (n)	16	3	
LIR	1.13 ± 0.15	1.03 ± 0.11	.145
Lymphedema severity grade			
Mild (n)	27	6	
Moderate (n)	0	0	
Severe (n)	0	0	
Radiotherapy			
Total volume (cGy)	5466.30 ± 303.58	5406.67 ± 261.28	.660
Cycles	19.04 ± 13.70	31.67 ± 2.16	.000*
Initial excess volume (mL)	107.64 ± 74.90	72.42 ± 55.62	.288
Reduction of excess volume after CDT (mL)	53.29 ± 45.75	16. 07 ± 26.75	.075

BMI = body mass index, CDT = complex decongestive therapy, LIR = lymphedema index ratio.

* *P* < .05.

The mean values of all patients were as follows: 55.42 ± 6.96 years of age, 1.58 ± 0.05 m in height, 61.74 ± 7.70 kg in weight, and a body mass index of 24.67 ± 2.86. The differences in volume were as follows: 101.24 ± 72.33 (mL) between the upper limbs, 1.06 ± 0.17 in the LIR, and 46.44 ± 45.00 (mL) before and after treatment in the affected upper limb. When the lymphedema severity grade was categorized using the ISL stages, all 33 patients were placed in Stage I (mild).

The primary outcomes are shown in Table 2. Lymphoscintigraphy of 8 patients (24.2%) showed no abnormal findings. Fifteen patients (45.5%) demonstrated a delayed lymphatic flow, while 6 patients (18.2%) showed no lymphatic flow. Eight patients (24.2%) were found to have a dermal back flow, and ALNU was absent in 11 patients (33.3%). A collateral pathway was absent in 21 patients (63.6%).

**Table 2:** Axillary lymph node uptake, lymphatic flow, dermal back flow, and the presence of any collateral pathway upon lymphoscintigraphy at each group.

	Group A (27, 81.8%)			Group B (6, 18.2%)			*P* value
Axillary lymph node uptake	Yes	Not detectable		Yes	Not detectable		.015*
	21 (77.8%)	6 (22.2%)		1 (16.7%)	5 (83.3%)		
Lymphatic flow	Normal	Delayed	Not detectable	Normal	Delayed	Not detectable	.034*
	12 (44.4%)	12 (44.4%)	3 (11.1%)	0 (0%)	3 (50%)	3 (50%)	
Dermal backflow	Yes	Not detectable		Yes	Not detectable		.733
	7 (25.9%)	20 (74.1%)		1 (16.7%)	5 (83.3%)		
Collateral pathway	Yes	Not detectable		Yes	Not detectable		.910
	10 (37.1%)	17 (62.9%)		2 (33.3%)	4 (66.7%)		

Patients who underwent SLND without radiation therapy at axillary site are in Group A. Patients who underwent SLND with radiation therapy at axillary site are in Group B. We found a significant difference (*P* value < .05) between groups at axillary lymph node uptake and lymphatic flow delay. However, there was no significant difference between groups at the dermal backflow and the presence of a collateral pathway (*P* value > .05).

SLND = sentinel lymph node dissection.

* *P* < .05.

In Group A, ALNU was not detectable in 6 patients (22.2%) (Table 2). Lymphatic flow was delayed in 12 patients (44.4%) and not detectable in 3 patients (11.1%) (Table 2). Dermal backflow was observed in 7 patients (25.9%) (Table 2). A collateral pathway was not observed in 17 patients (62.9%) (Table 2). In Group B, ALNU was not detectable in 5 patients (83.3%) (Table 2). Lymphatic flow was delayed in 3 patients (50%) and not detectable in 3 patients (50%) (Table 2). Dermal backflow was observed in 1 patient (16.7%) (Table 2). A collateral pathway was not observed in 4 patients (66.7%) (Table 2).

Significant differences (*P* value < .05) were confirmed in the presence of ALNU and lymphatic flow delay between the 2 groups using the Mann–Whitney *U* test and Kruskal–Wallis test (Table 2). However, there was no significant difference in the presence or absence of dermal backflow and the presence of a collateral pathway (*P* value > .05) (Table 2).

The initial excess volume(ml) was 107.64 ± 74.90 in group A, and 72.42 ± 55.62 in group B. The reduction of excess volume after CDT (mL) was 53.29 ± 45.75 in group A, and 16. 07 ± 26.75 in group B. There were no statistically significant differences in the secondary outcomes between the 2 groups.

## 4. Discussion

In the lymphoscintigraphy of all patients in this study, 84.8% (28 patients) had at least 1 abnormal finding (ALNU, lymphatic flow, dermal back flow, or a collateral pathway). When we analyzed the imaging characteristics of lymphoscintigraphy in patients diagnosed with BCRL after SLND by dividing them into groups of patients with or without aRTx, we found that there was a significant difference (*P* value < .05) in abnormal lymphoscintigraphy findings for ALNU and lymphatic flow delay between the 2 groups.

BCRL is a chronic disease that has many adverse effects on patients’ lives.^[14]^ Allison B et al collected information on the physical condition, daily living activities, social functioning, and psychological functioning of 97 BCRL patients through questionnaires conducted annually over 7 years.^[14]^ They reported that it affects the quality of life of patients in all aspects of physical, social, and psychological functioning not only immediately after diagnosis but also throughout the rest of their lives.^[14]^ We believe that education on BCRL and the proper treatment of patients are important so that BCRL patients do not miss the treatment time.

In a previous large cohort study, the incidence of BCRL after ALND was reported as up to 49% (n = 128 of the total 263).^[15]^ The incidence of BCRL was reported to be 0% to 7% in breast cancer patients who underwent SLND, which is less invasive than ALND.^[5,16,17]^ Although the incidence of BCRL in breast cancer patients who underwent SLND is lower than that of ALND patients, mild symptoms should not be overlooked to prevent further complications, such as late lymphedema or worsening of symptoms.^[18]^

RTx for breast cancer has been emphasized for a long time because the rate of early diagnosis of breast cancer increases and life expectancy is prolonged due to higher survival rates.^[6]^ However, RTx in breast cancer patients can cause various complications, such as upper extremity edema, brachial plexopathy, and decreased arm mobility. Among them, edema of the upper extremity is the most common, and it is caused by surgical removal of the lymph node, fibrosis caused by RTx, secondary deep-vein thrombosis, or infection.^[6]^ In particular, it is known that the incidence of BCRL increases when RTx is additionally performed after surgery.^[6,19,20]^ David Larson et al reported that in a study of 476 breast cancer patients, the incidence of BCRL was 4% when RTx alone was used while 13% when RTx was performed after ALND.^[19]^ Although lymphoscintigraphy still lacks common criteria and standardization for interpretation.,^[21]^ we suggest that the radiologic characteristics of lymphoscintigraphy are likely different according to whether the patient undergoes SLND, ALND, or RTx.

D.G. Lee et al investigated the severity of lymphedema by lymphoscintigraphy in 82 BCRL patients who underwent ALND.^[22]^ The severity of lymphedema as evaluated by arm circumference was not related to the type or extent of axillary lymph node dissection.^[22]^ Instead, the severity of lymphedema was worse in the group where ALNU was not observed on lymphoscintigraphy.^[22]^ In the present study, we found a statistically significant difference (*P* value < .05) between Groups A and B in abnormal lymphoscintigraphy findings for ALNU and lymphatic flow delays. Characteristically, in Group B, ALNU was not detectable in 83.3% of patients. In Group A, ALNU was not detectable in 22.2%. Based on these findings, we suggest that axillary lymph nodes may be affected not only by aRTx but also by SLND, which may lead to the observation of ALNU abnormalities on lymphoscintigraphy and may then further lead to the lymphedema onset and a poor prognosis.

Anatomically, the lymphatic system of the upper extremity starts from the lymph capillaries of the fingertip and palm and circulates through 20 to 40 axillary lymph node regions (sentry nodes).^[23]^ We hypothesized that the mechanical removal of the sentry node during the SLND procedure or fibrosis of the sentry node caused by RTx for breast cancer patients may have led to BCRL.

We reported normal findings on the lymphoscintigraphy of 24.2% of BCRL patients (Fig. 2). This result suggests that the possibility of BCRL should not be overlooked even if lymphoscintigraphy is normal when upper extremity edema is observed in a patient who underwent SLND. In addition, considering that all the patients in this study had a severity of Grade I (mild), BCRL would be more easily overlooked. In addition, an intensive early rehabilitation program after breast cancer surgery is known to be beneficial in reducing pain and edema in patients.^[24]^ Therefore, we should not miss the treatment timing of early lymphedema due to a lack of awareness of symptoms or the presence of mild symptoms (swelling, heaviness, and numbness, etc) in breast cancer patients. An evaluation of lymphedema through lymphoscintigraphy is essential; even if the lymphoscintigraphy is normal, BCRL should be diagnosed by synthesizing all the clinical signs. At the same time, education on BCRL should also be conducted in patients undergoing treatment for breast cancer.

There were 3 limitations to this study. First, there was a possibility of a selection bias because the sample size was small. Second, although all SLNDs were performed at the same hospital, various SLND methods that varied depending on the surgeon may have affected the lymphoscintigraphy and incidence of lymphedema. Third, patient follow-up after lymphoscintigraphy was not performed.

## 5. Conclusion

SLND by itself can affect the axillary lymph node activity, and the presence of aRTx also has a greater effect on the axillary lymph node activity and the lymphatic flow, which can be a risk factor for BCRL. However, although lymphoscintigraphy can be a clue for BCRL and abnormal findings can help determine the need for more aggressive therapeutic interventions in BCRL patients, considering 24.2% false negatives, clinicians should not overlook BCRL even if the lymphoscintigraphy is normal.

## Author contributions

**Conceptualization:** Jae Hyun Lee, Ghi Chan Kim, Ho Joong Jeong.

**Data curation:** Seung Tae Seong, Jun Young Park.

**Formal analysis:** Seung Tae Seong, Jun Young Park.

**Investigation:** Seung Tae Seong, Jun Young Park.

**Methodology:** Jae Hyun Lee, Ghi Chan Kim, Ho Joong Jeong.

**Writing – original draft:** Se Hyun Oh, Ju Hyeon Kim.

**Writing – review & editing:** Se Hyun Oh, Young Joo Sim.
